# Bioconcentration studies with the freshwater amphipod *Hyalella azteca*: are the results predictive of bioconcentration in fish?

**DOI:** 10.1007/s11356-018-3677-4

**Published:** 2018-11-16

**Authors:** Christian Schlechtriem, Sebastian Kampe, Hans-Jörg Bruckert, Ina Bischof, Ina Ebersbach, Verena Kosfeld, Matthias Kotthoff, Christoph Schäfers, Jacques L’Haridon

**Affiliations:** 1 Fraunhofer Institute for Molecular Biology and Applied Ecology, Auf dem Aberg 1, 57392 Schmallenberg, Germany; 2 L’Oréal Research & Innovation, Aulnay-sous-Bois, France

**Keywords:** Bioaccumulation, Alternative methods, Invertebrate, Freshwater amphipods, OECD 305, Flow-through test, Regulation

## Abstract

Bioconcentration factors (BCF) for regulatory purposes are usually determined by fish flow-through tests according to technical guidance document OECD 305. Fish bioconcentration studies are time consuming, expensive, and use many laboratory animals. The aim of this study was to investigate whether the freshwater amphipod *Hyalella azteca* can be used as an alternative test organism for bioconcentration studies. Fourteen substances of different hydrophobicity (log *K*_ow_ 2.4–7.6) were tested under flow-through conditions to determine steady state and kinetic bioconcentration factors (BCF_ss_ and BCF_k_). The results were compared with fish BCF estimates for the same substances described in the literature to show the relationship between both values. Bioconcentration studies with the freshwater amphipod *H. azteca* resulted in BCF estimates which show a strong correlation with fish BCF values (*r*^2^ = 0.69). *Hyalella* BCF values can be assessed in accordance with the regulatory B criterion (BCF > 2000, i.e., REACH) and thereby enable the prediction of B or non-B classification in the standard fish test. Therefore, *H. azteca* has a high potential to be used as alternative test organism to fish for bioconcentration studies.

**Electronic supplementary material:**

The online version of this article (10.1007/s11356-018-3677-4) contains supplementary material, which is available to authorized users.

## Introduction

The ultimate decisive bioaccumulation criterion as part of the regulatory chemical safety assessment of pesticides, biocides, pharmaceuticals, and other chemicals is the bioconcentration factor (BCF) expressing the potential of a test substance to be accumulated from the contaminated surrounding medium (European Commission 1998, 2009, 2012; VICH 2004). Bioconcentration factors (BCF) for regulatory purposes are usually determined by fish flow-through tests according to technical guidance document OECD 305 (OECD 2012). Fish bioconcentration studies are time consuming, expensive, and use many laboratory organisms in the range of 100–200 organisms per study. Alternative methods that may help to reduce the use of fish for BCF testing would therefore be of value.

The establishment of a new standard protocol for regulatory purposes requires a test organism which is constantly available, easy to handle in the laboratory, and has been successfully used in the past. *Hyalella azteca* is an epibenthic amphipod which is widespread in North and Middle America and commonly used for ecotoxicity studies with and without sediment (Environment Canada 2013; US EPA 2000; ASTM International 2000). The freshwater amphipods can be easily cultured in the laboratory and are available during the entire year. Due to their high reproduction rate and fast growth, experimental organisms can be raised within a few weeks to adult size to meet the need for a high amount of large organisms required for bioconcentration testing. In contrast to fish BCF tests, experimental organisms collected during the *Hyalella* test need to be pooled to provide sufficient biomass for tissue analysis. Several laboratory studies have been carried out with *H. azteca* to elucidate the bioconcentration potential of metals and organo-metals (Shuhaimi-Othman and Pascoe 2007; Norwood et al. 2007; Alves et al. 2009; Bartlett et al. 2004). Investigations on the toxicokinetics and bioconcentration of organic chemicals in *H. azteca* included chlorinated and polycyclic aromatic hydrocarbons, the insecticide DDT, and the synthetic hormone 17α-ethinylestradiol (Lee et al. 2002; Landrum et al. 2004; Nuutinen et al. 2003; Lotufo et al. 2000; Dussault et al. 2009). The water-only assays were usually carried out under static or semi-static conditions and did not follow a standardized protocol. BCF values for live amphipods measured at steady state (BCF_ss_) or calculated as the ratio of uptake and depuration rate constants (kinetic-based BCF values, BCF_kin_) are thus available. However, a systematic analysis of the potential of *H. azteca* as test organism for regulatory bioaccumulation studies has never been conducted.

The objective of this study was to estimate the bioconcentration potential of a wide range of substances in *H. azteca* to allow a comparison with fish BCF data described in the literature. For strongly hydrophobic substances (log *K*_ow_ > 5), testing via aqueous exposure may become increasingly difficult (e.g., due to sorption to the glass of exposure containers). Therefore, all tests were carried out under flow-through conditions in order to maintain aqueous concentrations at a level that is considered to be sufficiently constant.

Fourteen test substances of different hydrophobicity were applied including hexachlorobenzene (HCB); o-terphenyl (oTP); benzo(a)pyrene (BaP); pyrene, methoxychlor (MOCl); dibenz[a,h]anthracene; 1,2,3-trichlorobenzene; 2,4,5-trichlorophenol; PCB 153; PCB 77; diazinon, chlorpyrifos, simazine, and a further low hydrophobic compound (LHC) having a confidential structure. The correlation between fish and *Hyalella* BCF values was investigated to evaluate the potential of predicting bioconcentration in fish using a non-vertebrate species.

## Materials and methods

### Stock culture

The freshwater amphipod *H. azteca* used for the bioconcentration studies were raised in the laboratory of Fraunhofer IME, Schmallenberg. The strain was originally obtained from Freds Haustierzoo, Cologne, Germany. The stock culture was kept in 2-L flasks each stocked with 50 adult amphipods. Organisms were kept in reconstituted water containing bromide and were fed ground fish feed (Tetramin®) twice a week to maintain optimal growth (Environment Canada 2013). A small piece of gauze (3 × 3 cm) provided a place of refuge. Offspring were separated from the parent organisms once a week, placed in separate containers with a density of 150–200 juveniles per tank to be raised to culture size. After around 8 weeks, *H. azteca* reached maturity having a sufficient size to be used for bioconcentration studies. Care was taken that only healthy amphipods free from observable diseases and abnormalities were used in these studies. Male and female amphipods were usually separated to avoid reproduction during the experiment which may lead to the depuration of the previously accumulated test substance. However, the use of mixed groups including male and female amphipods was also tested. Males were distinguished by the presence of a large gnathopod. Female distinguishing characteristics include the absence of a gnathopod and presence of eggs in the marsupial plate.

### Bioconcentration studies

A 25-L glass aquarium filled with 20 L of test solution was used as test container and stocked with a group of around 1200 amphipods having a total weight of 1800–4140 mg depending on the type of animals used (male, female, or mixed). During the uptake phase of the flow-through tests lasting 2 to 12 days, the amphipods were continuously exposed to a constant concentration of the test substance provided at a flow rate of 2 to 12 L/h using a metering pump system (Table S1.1). Different flow rates were required to maintain stable exposure conditions. The concentration of the test substance in water was monitored throughout the uptake period to ensure constant exposure of the test organisms. In contrast to the aqueous exposure bioconcentration fish test (OECD 2012), at this time, a prediction of the length of the uptake phase and the time to steady state for the *Hyalella* BCF test cannot be made based on equations. As for fish, also for *H. azteca*, the duration of the uptake phase is obviously dependent on the hydrophobicity of the test substance with highly hydrophobic compounds requiring a longer time to reach steady state. Therefore, the exposure period was adjusted for each test chemical based on the experience from former studies with compounds of similar hydrophobicity to ensure that steady state will be reached.

**Fig. 1:**
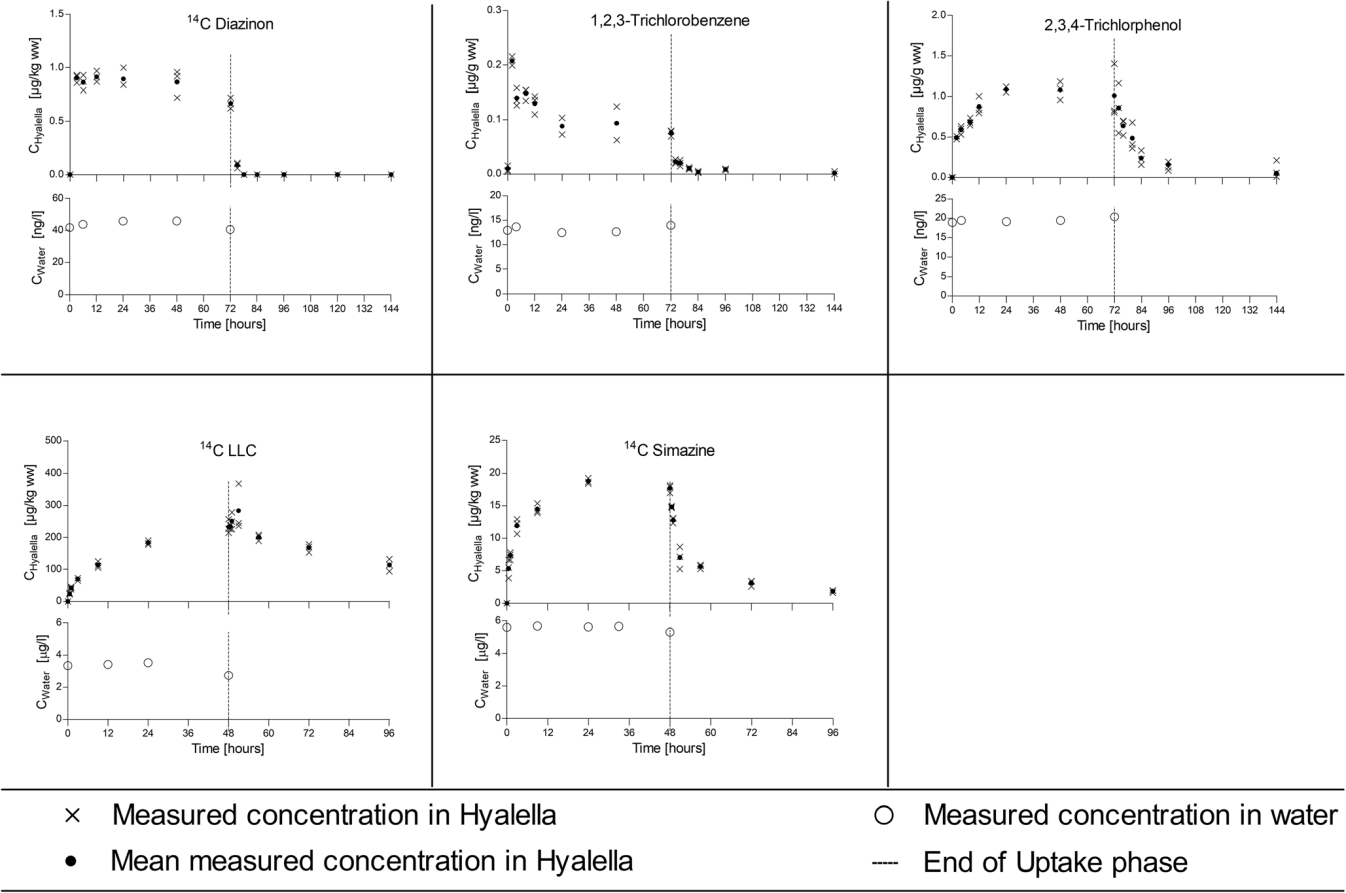
Bioconcentration experiments with male *H. azteca* on moderately or low lipophilic substances (log *K*_ow_ < 4). Each panel shows the time course of measured concentrations in the exposure water in the lower plot and the measured internal concentrations in the upper plot

**Fig. 2:**
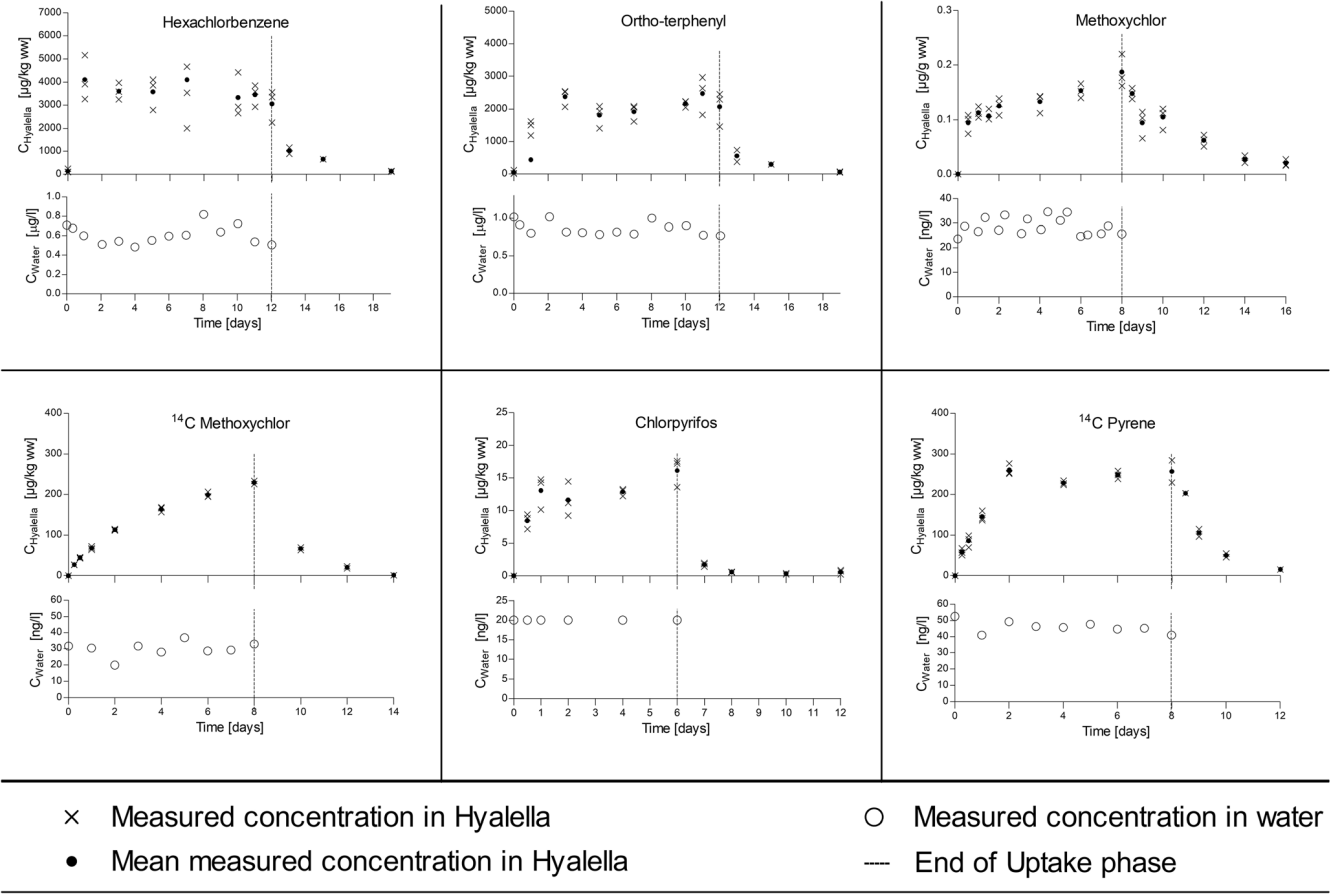
Bioconcentration experiments with male *H. azteca* on lipophilic substances (log *K*_ow_ of 4–6). Each panel shows the time course of measured* concentrations in the exposure water in the lower plot and the measured internal concentrations in the upper plot. * Nominal concentrations in water for chlorpyrifos

**Fig. 3:**
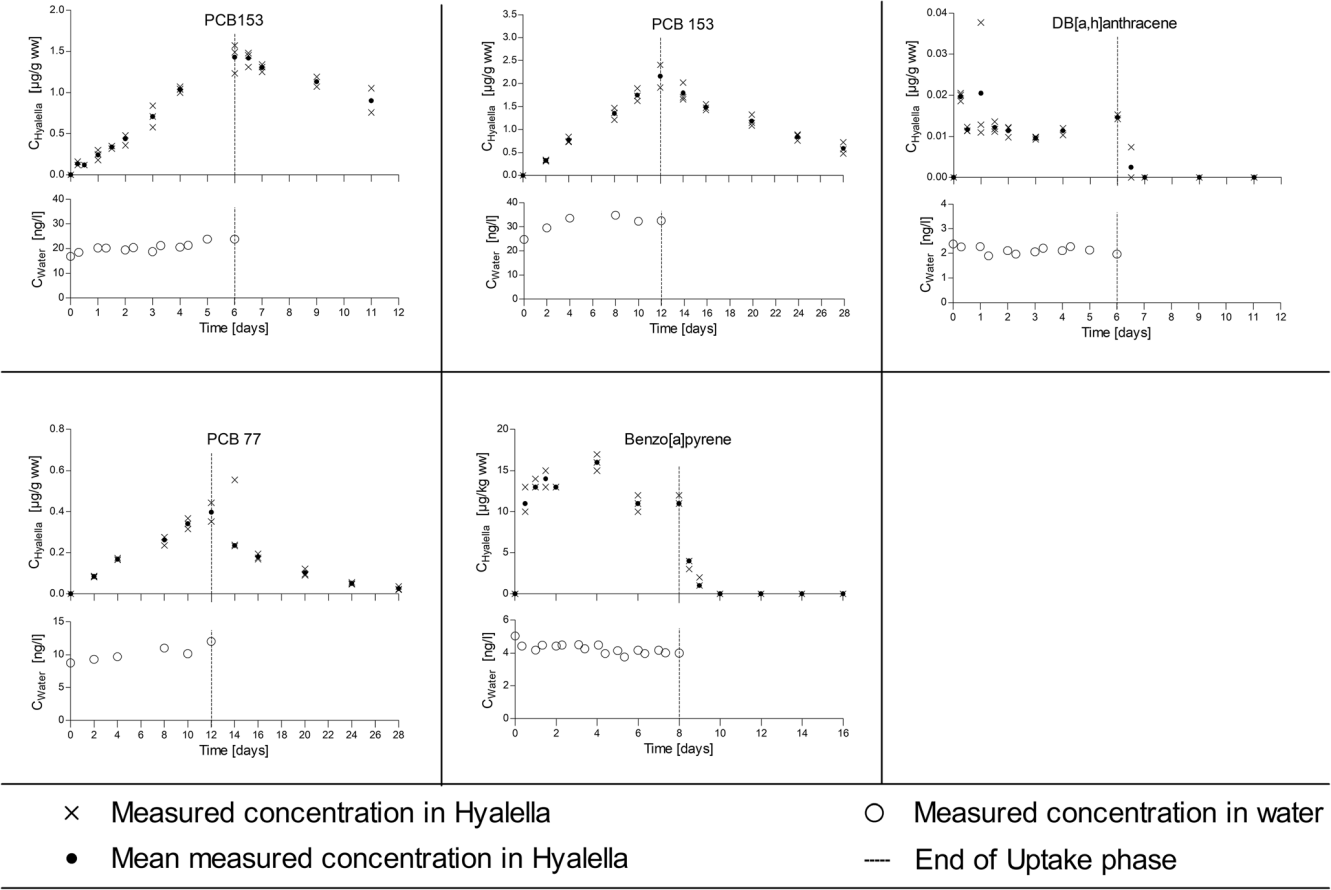
Bioconcentration experiments with male *H. azteca* on highly lipophilic substances (log *K*_ow_ > 6). Each panel shows the time course of measured concentrations in the exposure water in the lower plot and the measured internal concentrations in the upper plot

At the end of the uptake period, the amphipods were transferred into a new aquarium which had a continuous flow of clean dilution water to allow depuration of the previously accumulated test substance. The test chemicals and the length of the uptake and depuration periods applied in each study are described in Table 1. During the bioconcentration studies, amphipods were fed daily; algae aggregates (*Desmodesmus subspicatus*) using the filter disk method as described below. Emptied disks were removed from the experimental tank after feeding (between 30 min and 12 h, depending on feeding behavior) to keep the tanks as clean as possible. Amphipods were kept in a 16/8 h light/dark cycle throughout the study. Water temperature (23 ± 3 °C), pH (7.7–8.8), and dissolved oxygen concentrations (81–112%, 6.9–9.3 mg/L) were measured daily. The water in the test vessel was aerated via a glass capillary to maintain an oxygen level in the test system above 60% throughout the studies. Ammonia, nitrate, and nitrite were measured at the beginning and at the end of the uptake and depuration phases. All essential water quality parameters were constantly in a range acceptable for *H. azteca*. During the studies, samples of 3 times 20 amphipods were periodically removed from the test vessel, rinsed in dilution water, blotted dry, weighed (Shimadzu AUW220D), and immediately frozen at − 20 °C until chemical analysis. *Hyalella* and water samples were collected according to the schedule presented in Figs. 1, 2, and 3 and Fig. S1.1. Additional amphipods (3 × 10) were collected at the onset and the end of the uptake period for lipid analysis.

**Table 1:** Test substances, log *K*_ow_, uptake and depuration period, experimental organisms, and substance application in different bioconcentration tests in 20 L of test solution

Test	Test substance	Log *K*_ow_*	Uptake period (days)	Depuration period (days)	Males	Females	Mixed	Substance application**
I	Hexachlorobenzene	5.86	12	7	X	X		SP
I	Ortho-terphenyl	5.52	12	7	X	X		SP
II	PCB153	7.62	6	6	X	X		SP
II	Dibenz[a,h]anthracene	7.2	6	6	X	X		SP
III	Methoxychlor	5.67	8	8	X		X	SP
III	Benzo(a)pyrene	6.11	8	8	X		X	SP
IV	1,2,3-trichlorobenzene	3.93	3	3	X	X		SS
IV	2,4,5-trichlorphenol	3.45	3	3	X	X		SS
V	PCB153	7.62	12	14	X			SP
V	PCB77	6.34	12	14	X			SP
VI	Diazinon	3.86	3	3	X			SS
VII	Chlorpyrifos	4.66	6	6	X			SS
VIII	^14^C methoxychlor***	5.67	8	6	X			SS
IX	^14^C LHC***	3.36	2	2	X			SS
X	^14^C pyrene***	4.93	8	4	X			SS
XI	^14^C simazine***	2.4	2	2	X			SS

*EPI Suite (cited in Arnot and Gobas 2006); **SP, test solutions prepared with solid-phase desorption dosing system; SS, test solutions prepared from stock solutions. Further, information on substance application is provided as supporting information (Table S2). ***The specific radioactivity of the ^14^C radiolabelled test items was 8.19 MBq/mg (^14^C simazine), 5.17 MBq/mg (^14^C LHC), 12.71 MBq/mg (^14^C pyrene), and 32.18 MBq/mg (^14^C methoxychlor)

### Feeding of test organism

The unicellular green algae *Desmodesmus subspicatus* was obtained from SAG, culture collection of algae, Göttingen (Catalog No 86.81 SAG). The algae were cultured in growth medium according to Bringmann and Kühn (1980). After 6 days of incubation, algae were harvested by filtration through glass fiber filters (50 mm, Whatman GF 92). The algae-coated filters were frozen at − 20 °C prior to their use in the flow-through tests. Previous studies in our laboratory have shown that frozen aggregates of green algae are readily grazed from the filter surface by *H. azteca*. Green algae are of sufficient nutritional value to provide adequate nutrients during the experiment. Amphipods were fed ad libitum throughout the study; therefore, a feeding rate could not be determined.

The test system should be kept as clean as possible during bioconcentration studies (OECD, 305). Algae aggregates applied after storage at − 20 °C show a high stability in water. Once the filter surface has been grazed, the used filter disks with attached feed residues can be easily removed from the tank to keep the water in the test system as clean as possible.

### Test substances

Fourteen substances of different hydrophobicity (log *K*_ow_ 2.4–7.6) were tested in this study (Table 1). The range of substances included chlorinated diphenyls (PCB77; PCB153), a diphenylbenzene (o-terphenyl), a thiophosphoric acid ester derivative (diazinon), an organophosphate (chlorpyrifos), a triazine herbicide (simazine), polycyclic aromatic hydrocarbons (pyrene; benzo(a)pyrene; dibenz[a,h]anthracene), different organochlorine substances (hexachlorobenzene; methoxychlor; 2,4,5-trichlorophenol; 1,2,3-trichlorobenzene), and a further low hydrophobic compound (LHC). Some of the test substances were applied as ^14^C radiolabelled test substances (^14^C methoxychlor, ^14^C LHC, ^14^C pyrene, ^14^C simazine). Substances applied during the same test were dosed as a mixture (Table 1).

### Preparation of test solutions

Purified drinking water fulfilling the requirements defined by OECD 305 was used to prepare test solutions. The purification procedure included filtration with charcoal, aeration, and passage through a lime stone column. Test solutions of the highly hydrophobic test substances were obtained by means of a solid-phase desorption dosing system (Schlechtriem et al. 2017). The column-generated test substance concentrations were directed into a mixing chamber with magnetic stirring. Purified drinking water was added to the mixing chamber to reach the test concentration. Test solutions of the less hydrophobic test substances were prepared by dilution of stock solutions. Flow rates were between 2 and 12 L/h (Table S1.1). Pre-tests were carried out to exclude toxic effects of the concentrations used in the bioconcentration experiments.

### Chemical analysis

Hexachlorobenzene; *o*-terphenyl; PCB153; dibenz[a,h]anthracene; methoxychlor; benzo(a)pyrene; 1,2,3-trichlorobenzene; 2,4,5-trichlorophenol; and PCB77 were analyzed by gas chromatography (GC) coupled to mass spectrometry (MS), while diazinon and chlorpyrifos were analyzed with ultra-high performance liquid chromatography (UHPLC), coupled to a tandem mass spectrometer (MS/MS). GC-MS was performed on an Agilent 5973 Inert MSD equipped with an Rxi-5sil MS column (30 m, 0.25-mm ID, 0.25-μm film). Diazinon was analyzed on a Waters Xevo® TQD (Waters, USA) and chlorpyriphos on a Waters Xevo® TQ-S instrument (Waters, USA), equipped with a Waters BEH C18 UPLC column (100 × 5 mm, 1.7 μM). To assure analytical quality for all test substances internal standards were used as described in Table S1.2.

### Analysis of aqueous samples

Substances measured by GC were extracted by automated solid-phase microextraction (SPME) on polydimethyl siloxane fibers and injected by thermodesorption into the GC-MS instrument for analysis. However, 1,2,3-trichlorobenzene and 2,4,5-trichlorophenol were extracted with cyclohexane, and 2,4,5-trichlorophenol was derivatized with N-methyl-N-(trimethylsilyl) trifluoroacetamide (MSTFA) for 30 min at 65 °C before GC-MS analysis. Chlorpyrifos was extracted with methyl-*tert*-butylether (MTBE), dried under a nitrogen stream, redissolved in water/methanol (50/50), and measured by UHPLC-MS/MS. Aqueous diazinon samples were measured by UHPLC-MS/MS directly from solution after adding 200 μL of acetonitrile. Water samples containing ^14^C radiolabelled simazine, LHC, pyrene, and methoxychlor were analyzed for [^14^C] content by LSC (Tricarb TR/LL 2550, Packard Instruments, USA).

### Analysis of *Hyalella* samples

Pooled samples of 20 amphipods (about 40-mg fresh weight per sample) were homogenized with a B. Braun (Melsungen) homogenizer Potter (#853202). Substances analyzed by GC were extracted with dichloromethane/acetone (1:1) for 10 min in an ultrasonic bath and by vortex shaking, followed by centrifugation at 4000 rpm. The clear supernatants were transferred into a new tube, concentrated under a nitrogen stream to about 500 μL, and purified on silica SPE cartridges. Samples were eluted from the cartridges with dichloromethane/hexane (1:1) and transferred into sample vials where they were evaporated to dryness under a stream of nitrogen. After resolution in 250 μL toluene, the samples were analyzed by GC-MS. 1,2,3-trichlorobenzene and 2,4,5-trichlorophenol samples were redissolved in cyclohexane, the phenol derivatized with MSTFA for 30 min at 65 °C and both substances analyzed by GC-MS analysis. Chlorpyrifos was extracted with Methyl-*tert*-butylether (MTBE), dried under a stream of nitrogen, redissolved in water/methanol (50/50), and analyzed by UHPLC-MS/MS. *Hyalella* samples collected from the BCF study on diazinon were dried to dryness after silica SPE cleanup and redissolved in 500 μL acetonitrile, then 500 μL water was added. The suspension was agitated in an ultrasonic bath for 2 min, filtered over a syringe tip membrane filter (0.2 μm), and the clear solution taken and analyzed by LC-MS/MS.

Samples containing a radiolabelled substance were analyzed for [^14^C] content by combustion followed by LSC. Frozen samples were combusted in a biological oxidizer (OX500, Zinsser, Germany) at 900 °C for 3 min in the presence of 335 cc/min O_2_ and 335 cc/min N_2_. Radiolabeled CO_2_ was trapped in a scintillation cocktail (Oxysolve C-400, Zinsser Analytic, Germany) and quantified by LSC (Tricarb TR/LL 2550, Packard Instruments, USA).

### Determination of lipid content

Amphipods (3 × 10 animals) collected at the onset and the end of the uptake period were extracted by a slightly modified lipid extraction method originally described by Smedes and recommended by OECD 305 for gravimetric fish lipid determination (Smedes 1999; OECD 2012). Pooled samples of fresh amphipods were homogenized with 4.5 ml cyclohexan/isopropanol mix (5:4) by B. Braun (Melsungen) homogenizer Potter (#853202). Afterwards, 2.75 mL ultrapure water were added and the samples vortexed and then centrifuged for 12 min at 1650 rpm (396*g*). The organic phase was transferred into pre-weighed glass vials. Afterwards, 2.5 mL of cyclohexane/isopropanol (87%/13%) was added to the remaining aqueous phase. The samples were vortexed and centrifuged again. The organic phase was removed and pooled with the previously obtained fraction. The collected extract was evaporated under a stream of nitrogen and dried over night at 75 °C. Finally, the weight of the extracted lipids was determined (Mettler Toledo XP56) and the lipid content of the collected amphipods calculated on a fresh weight basis.

### Determination of test concentrations

Time-weighted average (TWA) concentrations of the test solutions were determined which account for the variation in concentration over time*.* First, weighted average concentrations were calculated by multiplying the average of two subsequently measured concentrations by the time period (h) between both measurements. All weighted average concentrations were then summed up and divided by the total time (h) of the uptake period resulting in the TWA concentration.

### Steady-state bioconcentration factor

A steady state was reached in the plot of test substance concentration in *Hyalella* (*C*_h_) against time when three successive analyses of *C*_h_ (μg/kg) made on samples taken at intervals of at least 2 days were within ± 20% of each other as described by OECD 305 (OECD 2012).

The steady-state BCF (BCF_SS_) was calculated as the quotient of the concentrations of the test substance in the *H. azteca* tissue (*C*_h_) in steady state and the corresponding TWA concentrations (μg/L) in the water (*C*_w_) according to Eq. 1:

1 $$ {\mathrm{BCF}}_{\mathrm{ss}}={C}_{\mathrm{h}}/{C}_{\mathrm{w}} $$

### Depuration rate constant

The depuration rate constant (*k*_2_) was calculated by fitting a one-compartment model to the measured concentrations in *Hyalella* during the depuration phase (Eq. 2):

2 $$ {C}_{\mathrm{h}\left(\mathrm{t}\right)}={C_{\mathrm{h}\left(\mathrm{t}\mathrm{i}\right)}}^{\ast }{e}^{\left(-\mathrm{k}2\ast \mathrm{t}\right)} $$

*C*_h(t)_: concentration in *H. azteca* at sampling (μg/kg).
*C*_h(ti)_: concentration in *H. azteca* (μg/kg) at the start of depuration phase (= 100%).

For the fitting, the concentrations were log_e_ transformed to allow linear regression of log concentrations versus time.

### Uptake rate constant

The uptake rate constant (*k*_1_) was calculated by non-linear regression analysis of the ratios *C*_h_/*C*_w_ against time during the uptake phase and including the depuration rate *k*_2_ fitted before. The fitted model assumes an attenuation of uptake by simultaneous elimination, increasing with increasing *C*_h_ up to equilibrium between uptake and elimination according to Eq. 3:

3 $$ {C}_{\mathrm{h}}/{C}_{\mathrm{w}}={k}_1/{k_2}^{\ast}\left(1-{\exp}^{\left(-\mathrm{k}2\ast \mathrm{t}\right)}\right) $$

### Kinetic bioconcentration factor

The kinetic bioconcentration factor (BCF_k_) was calculated by Eq. 4:

4 $$ {\mathrm{BCF}}_k={k}_1/{k}_2 $$

### Minimized design

BCF estimates were recalculated following a minimized design assuming that only one time point, tissue concentration at the end of uptake period, is available for the calculation of *k*_1_. The following formula (Eq. 5) was applied:

5 $$ {k}_{1\min }=\left({C_{\mathrm{h}}}^{\ast }{k}_2\right)/\left({C_{\mathrm{w}}}^{\ast}\left(1-{\exp}^{\left(-\mathrm{k}2\ast \mathrm{t}\right)}\right)\right) $$

The minimized kinetic bioconcentration factor (BCF_kmin_) was calculated by Eq. 6:

6 $$ {\mathrm{BCF}}_{\mathrm{kmin}}={k}_{1\min }/{k}_2 $$

Steady state and kinetic BCF estimates are in accordance with the standard fish test (OECD 2012). BCF_kmin_ were calculated to show that the uptake phase could be simplified. In contrast to OECD 305, depuration rate constants which were calculated as for the standard BCF design, i.e., with all sampling points of the depuration phase, were used for BCF_kmin_ calculation.

### Lipid normalization

The BCFs were normalized to 5% lipid content to allow the comparison with fish BCFs described in the literature.

### Literature search

A literature search (see Electronic Supplementary Material, Part S2) was conducted to find BCF estimates for fish which allow an objective comparison with the results obtained in this study on *H. azteca*. The correlation between the fish and *Hyalella* BCF data for the 14 test substances tested in this study was determined in order to prove the potential of bioconcentration studies with *H. azteca* to predict bioconcentration (log BCF ≥ 3.3) in the standard fish test. In case several BCF values from standard fish tests were available in the literature for one substance, the arithmetic mean and standard deviation were calculated (Table S2.1).

### Statistical calculations

The trajectories of water and tissue concentrations were presented by GraphPad Prism 5.01 (GraphPad Software). All calculations were done using Microsoft® Office Excel 2010 for calculation of means and SigmaStat 3.5 (Systat) for the linear regression analysis. Liner regression analysis of kinetic BCFs estimated for male *H. azteca* and of fish BCF estimates was carried out for the full set of fish BCF data and data obtained for single species (rainbow trout, common carp, and guppy). The uncertainties of *Hyalella* BCF values were calculated by the general law of propagation of errors without consideration of covariance (Mandel 1984). To determine the uncertainty of BCF_SS_, this calculation was based on the standard deviations of water and tissue samples (fish tissue and *Hyalella*), whereas for BCF_K_, the standard error of the *k*_1_ and *k*_2_ constant was applied for the law of propagation of errors. The standard error of *k*_1_ was taken from SigmaStat curve fitting, and for *k*_2_, the standard error of the slope of the linear regression calculated by Excel LINEST function was used. When normalizing to the lipid fraction, the standard deviation of lipid fraction was included in the same way (error propagation law) to obtain the final uncertainties of lipid-normalized BCF values.

## Results

### Weight and lipid content

The mean fresh weight and lipid content of the experimental organisms used for the bioconcentration studies are presented in Tables 2 and 3. The smallest and largest groups of male amphipods used had a mean fresh weight of 1.69 and 3.43 mg fresh weight (FW)/organism respectively. The mean fresh weight of female and mixed groups ranged from 1.04- to 2.41 mg FW/organism. The mean lipid content of male and female *H. azteca* determined gravimetrically ranged from 0.81 to 4.29%/FW and 1.95 to 3.43%/FW, respectively. Female amphipods showed a higher variation in lipid content of replicated samples in comparison to male amphipods as presented in Fig. S1.2.

**Table 2:** Aqueous concentrations (TWA), male animals, fresh weight, lipid content, uptake and depuration rate constants, and bioconcentration factors (BCF) with uncertainty (u)

Test	Test substance	TWA (ng L^−1^)	Sex	Mean *Hyalella* fresh weight (mg)	Mean lipid (%) ± SD (%)	*k*_1_ ± SE (L kg^−1^ d^−1^)	*k*_1min_ (L kg^−1^ d^−1^)	k_2_ ± SE (d^−1^)	Log BCF_ss_ ± u (L kg^−1^)	Log BCF_k_ ± u (L kg^−1^)	Log BCF_kmin_ (L kg^−1^)
I	Hexachlorobenzene	601	Male	3.43	1.29 ± 0.10	2753 ± 356	2133	0.417 ± 0.064	4.32 ± 0.80	4.41 ± 0.95	4.29
I	Ortho-terphenyl	856	Male	3.43	1.29 ± 0.10	1217 ± 80	1131	0.465 ± 0.082	4.01 ± 0.53	4.01 ± 0.81	3.97
II	PCB153	21	Male	1.69	n.a.	14,172 ± 453	n.a.	0.092 ± 0.006	n.a.	5.19 ± 0.39*	n.a.
II	DB[a,h]anthracene	2	Male	1.69	n.a.	n.a.	n.a.	n.a.	n.a.	n.a.	n.a.
III	Methoxychlor	29	Male	3.22	2.50 ± 0.20	2120 ± 251	1976	0.271 ± 0.023	4.01 ± 0.73	4.19 ± 0.70	4.16
III	Benzo(a)pyrene	4	Male	3.22	2.50 ± 0.20	6659 ± 400	n.a.	2.043 ± 0.098	3.78 ± 0.81	3.81 ± 0.42	n.a.
IV	1,2,3-trichlorobenzene	12,960	Male	2.02	1.26 ± 0.24	11 ± 5	6	0.963 ± 0.318	1.42 ± 0.30	1.66 ± 0.99	1.38
IV	2,4,5-trichlorphenol	19,440	Male	2.02	1.26 ± 0.24	69 ± 10	52	0.944 ± 0.164	2.33 ± 0.45	2.46 ± 0.72	2.34
V	PCB153	32	Male	2.90	1.94 ± 0.21	7884 ± 3307	n.a.	0.079 ± 0.003	n.a.	5.41 ± 0.65	n.a.
V	PCB77	10	Male	2.90	1.94 ± 0.21	6618 ± 347	n.a.	0.164 ± 0.006	n.a.	5.01 ± 0.63	n.a.
VI	Diazinon	44	Male	2.51	1.46 ± 0.43	36 ± 9	23	1.520 ± 1.018	1.79 ± 0.59	1.91 ± 1.47	1.72
VII	Chlorpyrifos	20	Male	2.26	2.37 ± 0.25	434 ± 59	404	0.473 ± 0.247	3.15 ± 0.55	3.29 ± 1.81	3.25
VIII	^14^C methoxychlor	23	Male	3.20	n.a.	4950 ± 361	6195	0.798 ± 0.112	3.82 ± 0.71*	3.79 ± 0.60*	3.89*
IX	^14^C LHC	3270	Male	2.78	0.81 ± 0.01	2.4 ± 0.3	2	0.017 ± 0.002	n.a.	2.95 ± 0.64	2.90
X	^14^C pyrene	46	Male	3.19	n.a.	4193 ± 201	4019	0.714 ± 0.052	3.73 ± 0.21*	3.77 ± 0.33*	3.75*
XI	^14^C simazine	5610	Male	2.95	1.56 ± 0.26	0.2 ± 0.04	0	0.043 ± 0.008	0.99 ± 0.20	1.18 ± 0.38	1.08

*BCF*_*ss*_, steady-state BCF; *BCF*_*k*_, kinetic BCF; *BCF*_*kmin*_, kinetic BCF following minimized design (BCF estimates normalized to 5% lipid content); *TWA*, time-weighted average concentrations in test solution; *SD*, standard deviation; *SE*, standard error; *u*, uncertainty; *n.a.*, no data available; mean lipid content (*n* = 4–6) of samples collected at beginning and end of uptake period

*BCF values not lipid normalized

**Table 3:** Aqueous concentrations (TWA), female and mixed animals, fresh weight, lipid content, uptake and depuration rate constants, and bioconcentration factors (BCF) with uncertainty (u)

Test	Test substance	TWA (ng L^−1^)	Sex	Mean *Hyalella* fresh weight (mg)	Mean lipid (%) ± SD (%)	*k*_1_ ± SE (L kg^−1^ d^−1^)	*k*_1min_ (L kg^−1^ d^−1^)	*k*_2_ ± SE (d^−1^)	Log BCF_ss_ ± u (L kg^−1^)	Log BCF_k_ ± u (L kg^−1^)	Log BCF_kmin_ (L kg^−1^)
I	Hexachlorobenzene	580	Female	2.41	2.44 ± 0.78	2693 ± 367	2343	0.256 ± 0.039	4.21 ± 1.44	4.33 ± 1.65	4.28
I	Ortho-terphenyl	875	Female	2.41	2.44 ± 0.78	1746 ± 161	1601	0.310 ± 0.055	3.97 ± 1.33	4.06 ± 1.53	4.03
II	PCB153	21	Female	1.04	n.a.	13,121 ± 524	n.a.	0.049 ± 0.013	n.a.	5.43 ± 1.42*	n.a.
II	DB[a,h]anthracene	2	Female	1.04	n.a.	n.a.	n.a.	n.a.	3.86 ± 1.21*	n.a.	n.a.
III	Methoxychlor	28	Mixed	1.71	3.26 ± 0.39	2192 ± 357	1780	0.119 ± 0.025	4.09 ± 0.65	4.45 ± 1.08	4.36
III	Benzo(a)pyrene	4	Mixed	1.71	3.26 ± 0.39	4983 ± 403	n.a.	1.497 ± 0.040	3.65 ± 0.47	3.71 ± 0.54	n.a.
IV	1,2,3-trichlorobenzene	13,410	Female	1.50	1.82 ± 0.67	14 ± 4	9	1.471 ± 0.226	1.30 ± 0.51	1.42 ± 0.70	1.23
IV	2,4,5-trichlorphenol	19,550	Female	1.50	1.82 ± 0.67	63 ± 6	55	0.857 ± 0.209	2.17 ± 0.82	2.31 ± 1.04	2.25

*BCF*_*ss*_, steady-state BCF; *BCF*_*k*_, kinetic BCF; *BCF*_*kmin*_, kinetic BCF following minimized design (BCF estimates normalized to 5% lipid content); *TWA*, time-weighted average concentrations in test solution; *SD*, standard deviation; *SE*, standard error; *u*, uncertainty; *n.a.*, no data available; mean lipid content (*n* = 4–6) of samples collected at beginning and end of uptake period

*BCF values not lipid normalized

### Water and tissue concentrations

Aqueous concentrations of the fourteen test substances measured during the uptake phase of the different bioconcentration studies are presented in Figs. 1, 2, and 3 and Fig. S1.1. Time-weighted average (TWA) concentrations (Tables 2 and 3) ranged from 2.1 ng/L (DB[a,h]anthracene) to 19.55 μg/L (2,4,5-Trichlorphenol). The concentration of the test substances was always below the limit of solubility in water in accordance with OECD 305. The tissue concentrations measured in male and female/mixed amphipods during the flow-through tests are presented in Figs. 1, 2, and 3 and Fig. S1.1, respectively.

### Estimated parameters

The kinetic and steady-state bioconcentration factors with estimated uncertainties as well as the related uptake and depuration rates are presented in Tables 2 and 3. All BCF values were normalized to 5% lipid content. Log BCF_k_ estimates showed a wide range of estimates from 1.18 (^14^C simazine) to 5.4 (PCB153) and seem to be largely independent of the animals (male, female, mixed culture) used. BCF_k_ estimates were often higher than the related BCF_ss_ indicating that the uptake period was not sufficient to reach steady-state conditions (e.g., chlorpyrifos, methoxychlor, BaP). In a few cases (PCB153, PCB77), steady-state conditions were not reached at the end of the uptake period, and therefore, only kinetic BCF estimates could be derived. BCF calculation following the minimized design resulted in bioconcentration factors (BCF_min_) which were comparable to the actual steady state and kinetic BCF estimates.

### Literature search

The literature screening was mainly based on a data collection compiled by Arnot and Gobas (2006) and resulted in a set of fish BCF estimates from bioconcentration studies with a broad range of fish species. Corresponding fish BCF data for the organic chemicals tested in this study were used if they were considered to be of acceptable confidence for bioconcentration assessment. The data were further evaluated to identify studies which were carried out or generated according to OECD TG 305 or in which all parameters described are closely related/comparable to the guideline method. Studies were selected if essential criteria were fulfilled including: (I) the chemical concentrations in the water were measured during the exposure period, (II) exposure under flow-through conditions, (III) acceptable weight range of the experimental animals,(IV) whole body analysis of tissue concentrations, and (V) the reported average chemical concentration in the water was less than or equal to the selected aqueous solubility. Missing information regarding one of the essential criteria was leading to the exclusion of a study from the further evaluation. Scientific literature which was published after 2006 was screened for further BCF estimates. Selected data were reviewed according to the criteria described above. The number of available data varied from one BCF estimate (e.g., methoxychlor) to 56 BCF estimates (chlorpyrifos) (Table S2.2). A summary of the literature search is presented in Table S2.1. Narrow to broad ranges of fish BCF values were found leading to different standard deviations.

### Comparison of fish and *Hyalella* BCF estimates

**Fig. 4:**
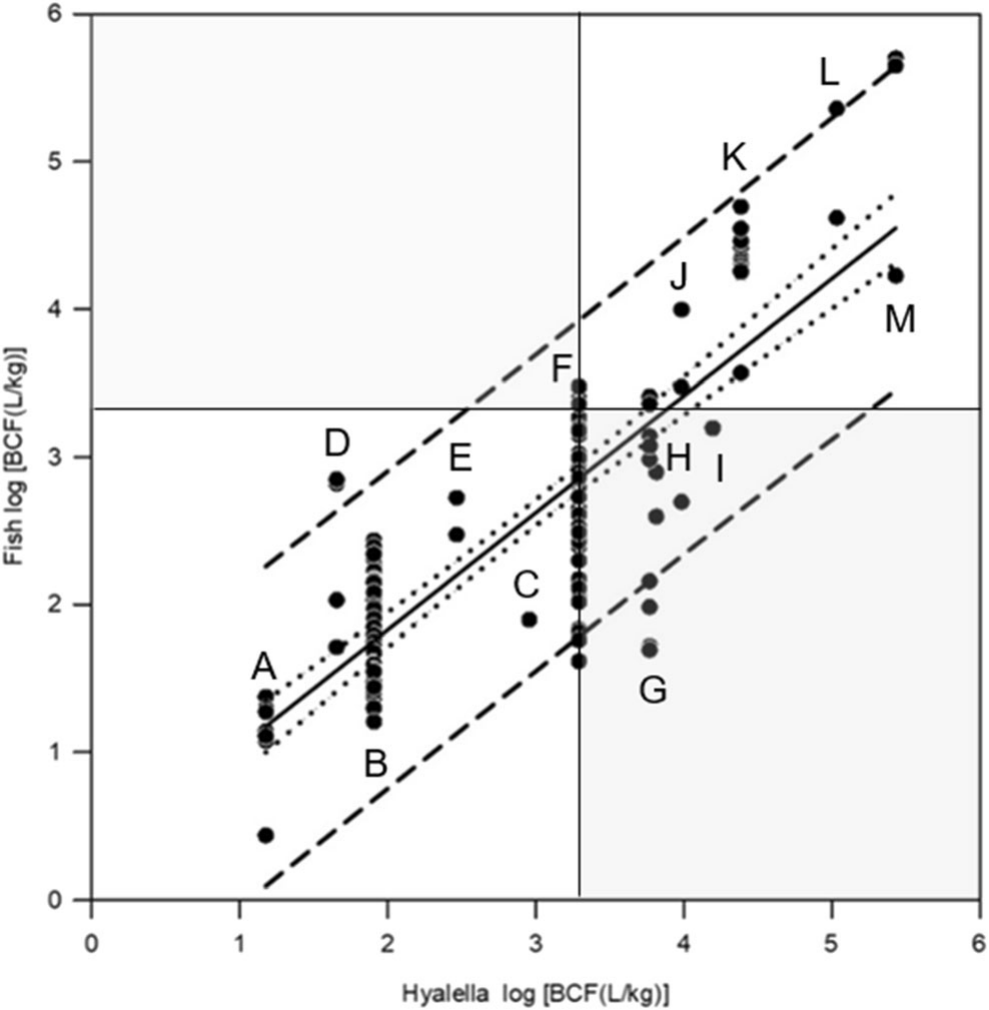
Experimental fish BCFs from different studies versus individual experimental kinetic BCFs estimated for male *Hyalella azteca* for thirteen chemicals with different log *K*_ow_. All *Hyalella* BCF values are normalized to 5% lipid content except for ^14^C-pyrene (G). The thin black lines mark the regulatory threshold of log BCF 3.3 (BCF 2000). Data points in the hatched area would relate to substances which highly accumulate in fish (log BCF ≥ 3.3) but not in *H. azteca* and vice versa representing type II and I error, respectively. Correlation: black regression line [fish log BCF = 0.251 + (0.792 × *Hyalella* log BCF)]; *R*^2^ = 0.687) with 95% confidence interval (dotted lines) and prediction interval (short dash). Standard error of the estimate (s_y׀x_) of the regression line = 1.1248. A, ^14^C-simazine; B, diazinon; C, ^14^C-low hydrophobic compound; D, 1,2,3-trichlorobenzene; E, 2,4,5-trichlorophenol; F, chlorpyrifos; G, ^14^C-pyrene; H, benzo(a)pyrene; I, methoxychlor; J, o-terphenyl; K, hexachlorobenzene; L, PCB77; M, PCB 153. References for fish BCF estimates are presented in Table S2.1. For detailed results of regression analysis see Electronic Supplementary Material, Part 3. A comparison of kinetic BCFs estimated for male *H. azteca* and fish BCF estimates for single species is presented in Figs. 5a–c

**Fig. 5:**
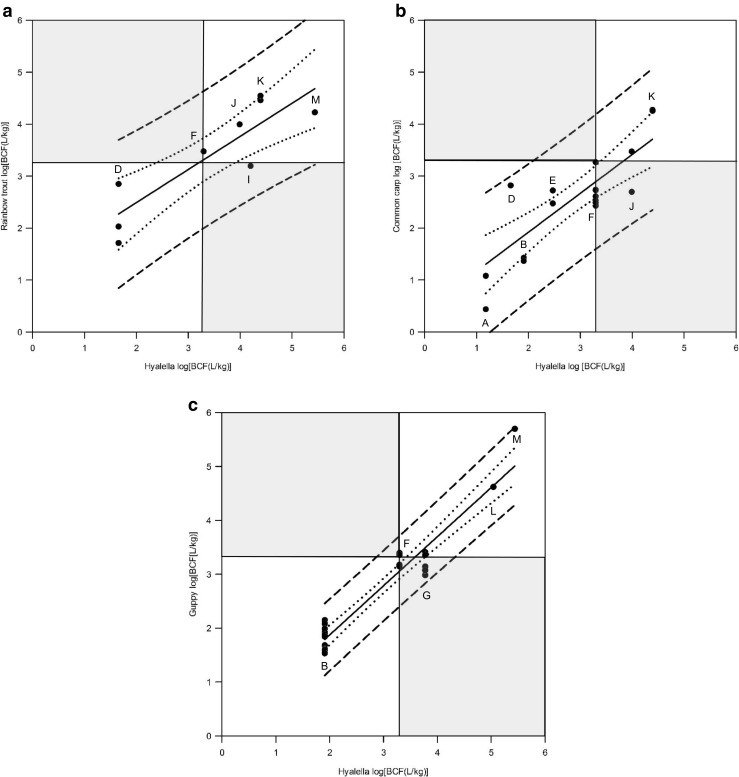
Comparison of kinetic BCFs estimated for male *H. azteca* and fish BCF estimates for rainbow trout (**a**), common carp (**b**), and guppy (**c**). *Hyalella* BCF values are normalized to 5% lipid content except for ^14^C pyrene (G). The thin black lines mark the regulatory threshold of log BCF 3.3 (BCF = 2000). Data points in the hatched area would relate to substances which highly accumulate in fish (log BCF ≥ 3.3) but not in *H. azteca* and vice versa representing type II error (upper left) and type I error (lower left), respectively. Black regression line with 95% confidence interval (dotted lines) and prediction interval (short dash). Test codes as defined in Fig. 4. For detailed results of linear regression see Electronic Supplementary Material Part 3

The relationship between *Hyalella* BCF values for thirteen of the tested chemicals and all fish BCFs collected from the literature is presented in Fig. 4. The linear regression resulted in a strong positive correlation (*r*^2^ = 0.69; see Electronic Supplementary Material, Part 3). The thin black lines in Fig. 4 mark the regulatory threshold of log BCF 3.3 (BCF 2000) applied in the PBT classification of chemical substances under the European REACH Regulation (European Commission 2011). Data points in the hatched upper left area of Fig. 4 would relate to substances which highly accumulate in fish (log BCF ≥ 3.3) but not in *H. azteca* (type II error*).* No data points are found in the hatched upper left area of Fig. 4. Experimental *Hyalella* BCF values tend to be higher compared to fish BCF estimates. Data points in the hatched lower right area of Fig. 4 would relate to substances which highly accumulate in *Hyalella* (log BCF ≥ 3.3) but not in fish (type I error*).* This was the case for ^14^C-pyrene, benzo(a)pyrene, and methoxychlor. When fish BCFs for single species were compared with the experimental *Hyalella* BCFs (Figs. 5a–c) linear regression resulted in higher correlation coefficients (e.g., rainbow trout and guppy) and smaller confidence and prediction intervals (e.g., guppy) compared to the total data set (see Electronic Supplementary Material, Part 3).

## Discussion

The results of this study demonstrate the suitability of bioconcentration tests with *H. azteca* to derive BCF estimates which are well established in the chemical regulatory system. Groups of 20 organisms turned out to be an adequate sample size to allow chemical analysis of the substances tested in this study. However, if more tissue material is required, the amount of amphipods pooled per sample can be increased accordingly. Considering all sample replicates (*n* = 3) and the series of sampling times required to estimate the kinetics of substance uptake and elimination, large-test populations of up to 1500 organisms may result. Following the minimized test design with only one time point, tissue concentration at the end of the uptake period could help to simplify the uptake phase and reduce the amount of test organisms required.

Populations of adult amphipods consist of male and female individuals. However, mixed test groups should be avoided to prevent the reproduction of the organisms during the study which would cause depuration of previously accumulated test substance by the release of juvenile amphipods.

The use of male amphipods facilitates the selection of homogeneous groups of experimental organisms and should be preferred to female organisms which tend to show a higher variability in size and body composition (lipid content) depending on their stage of reproduction. Female organisms are usually smaller than their male partners. Sexing of adult amphipods is easy based on a few characteristics such as female eggs and male claws.

Bioconcentration studies require the exposure to constant test concentrations. As shown in this study, flow-through bioconcentration tests with *H. azteca* can be carried out with low to high hydrophobic test substances. The solid-phase desorption dosing system helped to generate stable test concentrations of the test substances having a log *K*_ow_ > 5 (Schlechtriem et al. 2017).

Fish flow-through tests are commonly carried out in large aquariums with a volume of up to 100 L to reach a loading rate of 0.1–1.0 g of fish (fresh weight) per liter of water per day which is recommended to maintain adequate dissolved oxygen concentrations and minimize test organism stress (OECD 2012). The bioconcentration test with *H. azteca* enables reduction of the size of the test system due to the small size of the animals. Due to the shorter exposure period required to reach steady-state conditions and the comparatively lower media consumption, running flow-through tests with *H. azteca* can also lead to substantial savings of test substance compared to fish BCF tests. In this study, experimental tanks with a volume of 20 L were used to keep the experimental groups consisting of 1000 to 1200 amphipods. With regard to the small total biomass of the test organisms, the volume of the tanks could possibly be further reduced which may help to minimize the amount of test media required to run the flow-through test.

As shown in the literature, BCF tests with freshwater amphipods may also be carried out under static or semi-static exposure conditions at least with stable substances. Such tests may well result in similar results to those obtained by flow-through tests as shown by Lee et al. (2002) where a log BCF value of 3.7 was estimated for pyrene which is similar to the result obtained in this study (log BCF_k_ of 3.8). Schuytema et al. (1988) determined a BCF for HCB in a static test system with *H. azteca*. The concentration in the water was maintained by a gas-phase transfer method. The log BCF calculated after 28 days of exposure in flasks was 4.4 which is in agreement with the value obtained in this study (log BCF_k_ of 4.4). However, the large amount of test organisms required for BCF testing may result in a deterioration of water quality in static test systems and thus requires particular caution. Flow-through conditions as applied in this study help to maintain stable test concentrations and keep the water quality at a constant acceptable level.

During the bioconcentration test *H. azteca* may shed their skin and discard their “molt” which can be removed from the water surface. It cannot be avoided that amphipods which die during the experiment are eaten by their siblings even if this is in contradiction to the findings of a former study by Hargrave (1970). However, the uptake of test chemicals by ingestion of dead organisms should be negligible in comparison to the uptake by bioconcentration processes.

As a result of this study, steady state and/or kinetic BCF estimates were calculated for all test substances. For several test substances, kinetic and steady-state BCF estimates were comparable proving that organisms were exposed for a sufficient time to reach steady-state conditions. For highly hydrophobic substances like PCB 153 and PCB77, only kinetic BCF could be determined due to the limited uptake period. Comparing the hydrophobicity (octanol/water partition coefficient, log *K*_ow_) of the test substances and the time required to reach steady-state conditions, a general recommendation can be inferred as follows. For moderately or low hydrophobic substances (log *K*_ow_ < 4), 2 days seem to be a sufficient exposure period. Hydrophobic substances (log *K*_ow_ of 4–6) should be exposed at least for 4 days to ensure that steady-state conditions are reached at the end of the uptake period. For highly hydrophobic substances (log *K*_ow_ > 6) such as PCB153 exposure periods lasting more than 12 days seem required. In this last case, the calculation of BCF_ss_ should be replaced by the kinetic BCF to avoid a further extension of the uptake period. Generally, the exposure period should be kept as short as possible to ensure optimal conditions of the experimental organisms. As shown in this study, the *Hyalella* flow-through test can be further simplified by using a minimized aqueous exposure test setup with fewer sampling points which allows a reduction in the number of organisms and/or resources (OECD 2012; Springer et al. 2008).

In this study, only lipid accumulating substances which tend to associate with hydrophobic tissues were tested. Lipids in *H. azteca* are mainly deposited in lipid droplets adjacent to the gut and in the lipid-rich nervous tissues of the ventral segmental ganglia and protocerebrum. As in the fish, triacylglycerols represent the most abundant lipid class in *H. azteca* (Arts et al. 1995). The lipid content in *H. azteca* may vary depending on the size and age of the amphipods and tends to be lower compared to the lipid levels measured in fish used for bioconcentration testing. Therefore, lipid normalization of the estimated BCF values was required to allow the comparison with BCF estimates from fish studies. Lipid normalization to a lipid level of 5% was carried out as recommended by OECD 305.

BCF values calculated for *H. azteca* tended to be higher compared to fish but were still showing a clear correlation with the fish BCF estimates. Contrasting BCF values might be explained by differences in the bioconcentration kinetics. A few studies have investigated the uptake, biotransformation, and depuration rates for contaminants in *H. azteca*. Biotransformation processes (generally classified as phase I and phase II reactions) can be a key factor affecting bioconcentration. The toxicokinetics of polycyclic aromatic hydrocarbons (PAH) in *H. azteca* was investigated by Lee et al. (2002). A two-compartment model that included biotransformation was applied to describe the kinetics of pentachlorophenol, methyl parathion, fluoranthene, and 2,2′,4,4′,5,5′-hexachlorobiphenyl in *H. azteca* (Nuutinen et al. 2003). *H. azteca* has the ability to metabolize substances with varying chemical structures. The metabolism of anthracene, fluoranthene, DDT, and 2,4,6-trinitrotoluene was investigated (Landrum and Scavia 1983; Kane Dristoll et al. 1997; Lotufo et al. 2000; Sims and Steevens 2008). General biotransformation pathways in freshwater crustaceans have been described by Katagi and Whitacre (2010) and Jeon et al. (2013). Certain metabolic pathways (e.g., glucuronidation) are obviously not present in freshwater crustaceans*.* The limited biotransformation capacity of the amphipods may explain why BCF values calculated for *H. azteca* tended to be higher compared to fish. Additional investigations are required to further elucidate the metabolism of xenobiotic substances in *H. azteca*, to identify species-specific metabolites, and to assess the impact of biotransformation processes on the outcome of bioconcentration studies.

The fish BCF data collection described by Arnot and Gobas (2006) shows that BCF data even from single research groups can have a considerable variation leading to a significant scatter of the available BCF data. The scatter may come from the use of different fish species with possibly different metabolic rates, different fish sizes, and factors that are not strictly standardized in current BCF tests. Despite the scatter, a clear correlation between *Hyalella* and fish BCF estimates was observed. It was investigated whether the results of *Hyalella* bioconcentration studies are predictive of bioconcentration in fish without leading to false conclusions. In this context, the question whether a chemical may highly accumulate in fish (BCF > 2000, i.e., REACH) but not in *H. azteca* (type II error) resulting in a non-B classification was of particular concern. For none of the substances tested in this study, a type II error was obtained. Whenever log BCF was < 3.3 (BCF < 2000) for *Hyalella*, this was also the case for fish. However, prediction intervals for the full set of data clearly indicated that such a scenario may still occur with a certain probability, given what has already been observed. Due to the high scatter of fish BCF data, that is, highly problematic from a regulatory point of view, unambiguous predictions cannot be expected and strict standardization is recommended. As shown in this study, the comparison of kinetic BCFs estimated for male *H. azteca* and fish BCF estimates for single species may already significantly reduce the uncertainty in BCF prediction. The comparison of *Hyalella* BCF values with guppy BCF data resulted in a very high correlation coefficient (*R*^2^ = 0.92) and comparably small confidence and prediction intervals which might be explained by the greater homogeneity of the small test animals compared to common carp and rainbow trout. Additional *Hyalella* BCF studies should be carried out to further improve the linear regression models based on extended data sets allowing to predict fish BCF values while keeping the type II error as low as possible. However, also the performance of *Hyalella* BCF tests should be strictly standardized to reduce error in the measured BCFs. Selection of homogenous test populations and accurate determination of lipid contents for lipid normalization are central requirements (Schlechtriem et al. 2012).

BCF values calculated for *H. azteca* tend to be higher compared to fish leading to a type I error falsely inferring the existence of a high bioaccumulation potential for a chemical in fish (BCF > 2000) that is not there. “False positive” findings are of minor concern from a regulatory perspective but should still allow for an appropriate assessment based on predicted fish BCF estimates.

In conclusion, bioconcentration studies with the freshwater amphipod *H. azteca* result in BCF estimates which show a strong correlation with fish BCF values. Therefore, *H. azteca* has a high potential to be used as alternative test organism to fish for bioconcentration studies. So far, only lipid accumulating substances have been tested with *H. azteca*. Further studies are required to elucidate the bioconcentration of non-lipid accumulating substances.

## Electronic supplementary material

ESM 1 (DOCX 352 kb)
