# Supplementary material for: Bioconcentration studies with the freshwater amphipod Hyalella azteca: are the results predictive of bioconcentration in fish?
Source: Environ Sci Pollut Res Int. 2018 Nov 16;26(2):1628–41. doi: 10.1007/s11356-018-3677-4 (PMC6331748; doi:10.1007/s11356-018-3677-4)
Supplement: Supplementary file 1 — (DOCX 352 kb) [file 11356_2018_3677_MOESM1_ESM.docx]

**Supplementary material**

**Bioconcentration studies with the freshwater amphipod *Hyalella azteca*. Are the results predictive of bioconcentration in fish?**

Christian Schlechtriem †*, Sebastian Kampe †, Hans-Jörg Bruckert †, Ina Bischof †, Ina Ebersbach †, Verena Kosfeld †, Matthias Kotthoff †, Christoph Schäfers †, and Jacques L’Haridon ‡

^†^ Fraunhofer Institute for Molecular Biology and Applied Ecology, Auf dem Aberg 1, 57392 Schmallenberg, Germany

‡ L´Oréal Research & Innovation, Aulnay-sous-Bois, France

*Environmental Science and Pollution Research*

**Supplementary material (Part 1)**

**S1. Information on experimental procedures, test animals and chemical analysis**

Table S1.1 Preparation of test solutions

| Test | Test substance | Substance application** | No. of columns | Loading of carrier material (µg/g) | Flow rate into column (mL/min) | Total flow rate (L/h) |
| --- | --- | --- | --- | --- | --- | --- |
| I | hexachlorobenzene | SP | 1 | 1000 | 15 | 8.1 |
| I | o-terphenyl | SP | 1 | 4000 | 1 | 8.1 |
| II | PCB153 | SP | 1 | 200 | 6 | 6.2 |
| II | dibenz[a,h]anthracene | SP | 1 | 180 | 6 | 6.2 |
| III | methoxychlor | SP | 1 | 400 | 5 | 6.4 |
| III | benzo(a)pyrene | SP | 1 | 200 | 17.5 | 6.4 |
| IV | 1,2,3-trichlorobenzene | SS |  |  |  | 7.8 |
| IV | 2,4,5-trichlorphenol | SS |  |  |  | 7.8 |
| V | PCB153 | SP | 1 | 200 | 5 | 7.2 |
| V | PCB77 | SP | 1 | 200 | 15 | 7.2 |
| VI | diazinon | SS |  |  |  | 12.0 |
| VII | chlorpyrifos | SS |  |  |  | 12.0 |
| VIII | ^14^C methoxychlor | SS |  |  |  | 6.0 |
| IX | ^14^C LHC | SS |  |  |  | 6.0 |
| X | ^14^C pyrene | SS |  |  |  | 6.0 |
| XI | ^14^C simazine | SS |  |  |  | 2.0 |

******SP, test solutions prepared with solid-phase desorption dosing system; SS, test solutions prepared from stock solutions

Table S1.2 Masses (GC-MS), Mass transitions (LC-MS/MS), internal standards and SPE cartridges used for chemical analysis of water and amphipod samples.

| **Analyte** | **Analytical technique** | **Internal standard** | **SPE cartridges used** | **m/z*** |
| --- | --- | --- | --- | --- |
| hexachlorobenzene | GC-MS | hexachlorobenzene ^13^C_6_ | Sep-Pak Silica gel (Waters) | **184**, 249 |
| *o*-terphenyl | GC-MS | *o*-terphenyl d_14_ |  | **230**, 215 |
| PCB153 | GC-MS | PCB138 |  | **359.8**, 361.8 |
| dibenz[a,h]anthracene | GC-MS | dibenz[a,h]anthracene d_14_ |  | **278**🠒 **97**🠒197.9 |
| PCB77 | GC-MS | PCB 138 |  | **291.9**, 289.9 |
| methoxychlor | GC-MS | 4,4’-DDE (dichlorodiphenyl-dichloroethylene) |  | **229**, 274 |
| benzo(a)pyrene | GC-MS | benzo(a)pyrene -d_12_ |  | **252**, 253 |
| 1,2,3-trichlorobenzene | GC-MS | 1,2,4-trichlorobenzene | Strata SI-1 Silica (Phenomenex) | **179.9** |
| 2,4,5-trichlorophenol  (as TMS derivative) | GC-MS | 2,3,4-trichlorophenol (as TMS derivative) |  | **252.9** |
| diazinon | LC-MS/MS | No IS | Sep-Pak Silica gel (Waters) | 305.1 🠒 **169.0**🠒96.9 |
| chlorpyrifos | LC-MS/MS | chlorpyrifos -d_10_ | - | 349.9 🠒 **97**🠒197.9 |

* In bold, the masses/transitions used for quantification of analytical results.

Figure S1.1. Bioconcentration experiments with female/mixed *H. azteca* on moderately to highly lipophilic substances. Each panel shows the time course of measured concentrations in the exposure water in the lower plot and the measured internal concentrations in the upper plot. Mixed cultures are indicated with an asterisk*


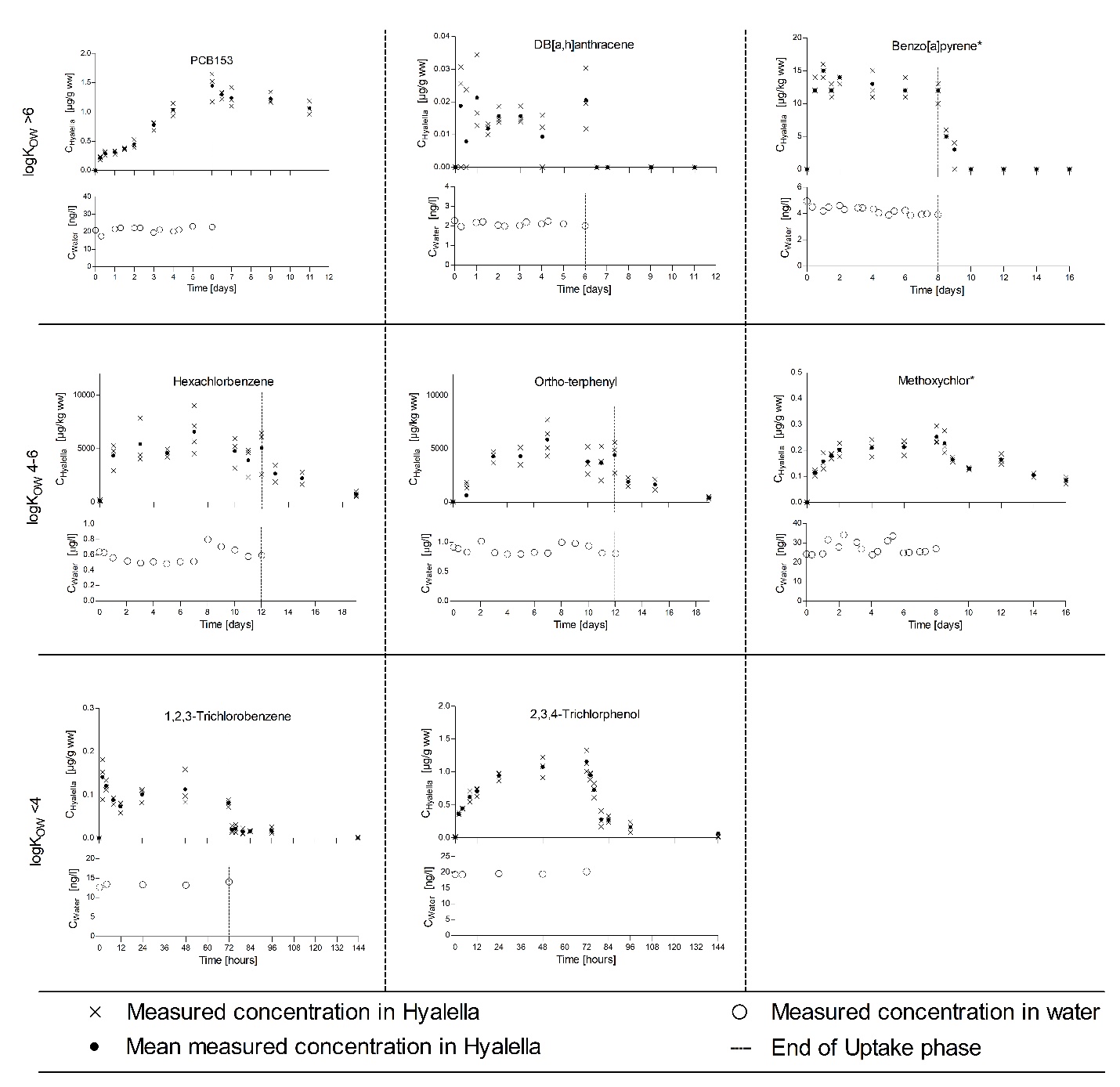


Figure S1.2 Lipid content (% of FW) of male (black diamonds) and female (grey squares) *H. azteca* used for flow-through BCF tests (start of uptake). Mean values ± SD (n=4-6); Test codes as defined in Table S1.1.


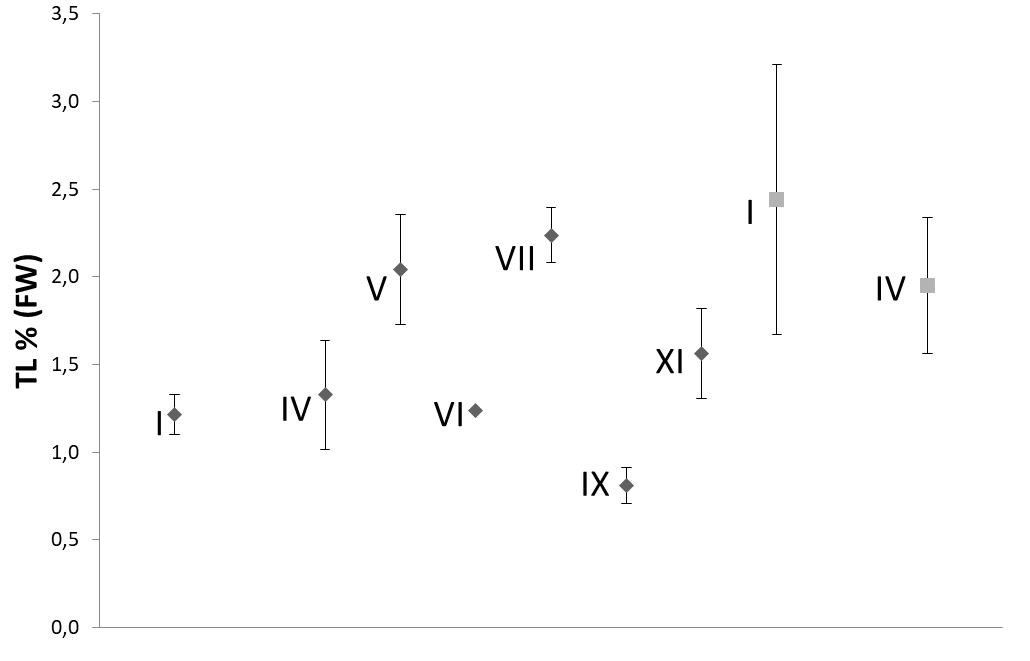


**Supplementary material (Part 2)**

**S2.1 Literature search on fish BCF estimates**

A literature search was carried out based on the BCF data collection presented by Arnot and Gobas (2006) to find fish BCF estimates which allow a comparison with the results obtained in this study on *H. azteca*. Results of the literature search are presented in Tables S2.1 and S2.2.

**Table S2.1 Summary of literature search**

| Label of substances (Figure 4) | Chemical CAS# | Test substances | log K_ow_* | References | Mean Fish log BCF | ±SD |
| --- | --- | --- | --- | --- | --- | --- |
| A | 122-34-9 | ^14^C simazine | 2.4 | CERI 1992; Tsuda et al. 1992 | 1.1 | 0.3 |
| B | 333-41-5 | diazinon | 3.86 | CERI 1992; Kanazawa 1978, Tsuda et al. 1995; Tsuda et al. 1997 | 1.8 | 0.3 |
| C | nd | ^14^C- LHC | 3.36 | Schlechtriem et al. 2016 | 1.9 |  |
| D | 87-61-6 | 1,2,3-trichlorobenzene | 3.93 | CERI 1992; Galassi & Calamari 1982 | 2.4 | 0.5 |
| E | 95-95-4 | 2,4,5-trichlorophenol | 3.45 | CERI 1992 | 2.6 | 0.2 |
| F | 2921-88-2 | chlorpyrifos | 4.66 | Adolfsson-Erici et al. 2012; Cripe et al. 1986; Goodman et al. 1985a; Goodman et al. 1985b; Jarvinen et al. 1983, NITE 2005; Tsuda et al. 1992; Tsuda et al. 1997 | 2.7 | 0.5 |
| G | 129-000-0 | ^14^C-pyrene | 4.93 | Carlson et al. 1979; Jonsson et al. 2004 | 2.7 | 0.7 |
| H | 50-32-8 | benzo(a)pyrene | 6.11 | Jimenez et al. 1987 | 2.8 | 0.2 |
| I | 72-43-5 | methoxychlor | 5.67 | Bischof 2018 | 3.2 |  |
| J | 84-15-1 | o-terphenyl | 5.52 | Böhm et al. 2017; CERI 1992 | 3.4 | 0.7 |
| K | 118-74-1 | hexachlorobenzene | 5.86 | Adolfsson-Erici et al. 2012; Böhm et al. 2017; Carlson & Kosian 1997; CERI 1992; Chaisukant et al. 1997; Kosian et al. 1981 | 4.3 | 0.3 |
| L | 32598-13-3 | PCB77 | 6.34 | Fox et al. 1994; Opperhuizen & Voors 1987 | 5 | 0.5 |
| M | 35065-27-1 | PCB153 | 7.62 | Böhm et al. 2017; Fox et al. 1994; Opperhuizen & Schrap 1987 | 5.2 | 0.8 |

* EPI Suite (cited in Arnot and Gobas 2006)

**Table S2.2 Results of literature search**

| Chemical | CAS # | Fish species | log BCF | Estimated | Reference |
| --- | --- | --- | --- | --- | --- |
|  |  |  | Exptl | Log K_ow_ |  |
|  |  |  | (L/kg) | EPISUITE |  |
| Hexachlorobenzene | 118741 | Fathead minnow | 4.7 | 5.86 | Kosian et al. 1981 |
| Hexachlorobenzene | 118741 | Fathead minnow | 4.4 | 5.86 | Carlson & Kosian 1987 |
| Hexachlorobenzene | 118741 | Fathead minnow | 4.4 | 5.86 | Carlson & Kosian 1987 |
| Hexachlorobenzene | 118741 | Fathead minnow | 4.3 | 5.86 | Carlson & Kosian 1987 |
| Hexachlorobenzene | 118741 | Fathead minnow | 4.3 | 5.86 | Carlson & Kosian 1987 |
| Hexachlorobenzene | 118741 | Fathead minnow | 4.2 | 5.86 | Carlson & Kosian 1987 |
| Hexachlorobenzene | 118741 | Western mosquitofish | 3.6 | 5.86 | Chaisuksant et al. 1997 |
| Hexachlorobenzene | 118741 | Common carp | 4.3 | 5.86 | CERI 1992 |
| Hexachlorobenzene | 118741 | Common carp | 4.3 | 5.86 | CERI 1992 |
| Hexachlorobenzene | 118741 | Rainbow trout | 4.5 | 5.86 | Böhm et al. 2017 |
| Hexachlorobenzene | 118741 | Rainbow trout | 4.6 | 5.86 | Adolfsson-Erici et al. 2012 |
| Ortho-terphenyl | 84151 | Common carp | 2.7 | 5.52 | CERI 1992 |
| Ortho-terphenyl | 84151 | Common carp | 3.5 | 5.52 | CERI 1992 |
| Ortho-terphenyl | 84151 | Rainbow trout | 4.0 | 5.52 | Böhm et al. 2017 |
| PCB153 | 35065271 | Guppy | 5.7 | 7.8 | Opperhuizen & Shrap 1987 |
| PCB153 | 35065271 | Rainbow trout | 4.2 | 7.8 | Böhm et al. 2017 |
| PCB153 | 35065271 | Zebrafish | 5.7 | 7.8 | Fox et al. 1994 |
| Benzo(a)pyrene | 50328 | Bluegill sunfish | 2.9 | 6.11 | Jimenez et al. 1987 |
| Benzo(a)pyrene | 50328 | Bluegill sunfish | 2.6 | 6.11 | Jimenez et al. 1987 |
| 1,2,3-Trichlorobenzene | 87616 | Common carp | 2.8 | 3.93 | CERI 1992 |
| 1,2,3-Trichlorobenzene | 87616 | Common carp | 2.8 | 3.93 | CERI 1992 |
| 1,2,3-Trichlorobenzene | 87616 | Rainbow trout | 2.9 | 3.93 | Galassi et al. 1983 |
| 1,2,3-Trichlorobenzene | 87616 | Rainbow trout | 2.0 | 3.93 | Galassi et al. 1983 |
| 1,2,3-Trichlorobenzene | 87616 | Rainbow trout | 1.7 | 3.93 | Galassi et al. 1983 |
| 2,4,5-Trichlorphenol | 95954 | Common carp | 2.7 | 3.45 | CERI 1992 |
| 2,4,5-Trichlorphenol | 95954 | Common carp | 2.5 | 3.45 | CERI 1992 |
| PCB77 | 32598133 | Guppy | 4.6 | 6.34 | Opperhuizen & Voors 1987 |
| PCB77 | 32598133 | Zebrafish | 5.4 | 6.34 | Fox et al. 1994 |
| Diazinon | 333415 | Biwi lake gudgeon, goby or willow shiner | 2.3 | 3.86 | Kanazawa, J. 1978 |
| Diazinon | 333415 | Common carp | 1.4 | 3.86 | Kanazawa, J. 1978 |
| Diazinon | 333415 | Common carp | 1.4 | 3.86 | Kanazawa, J. 1978 |
| Diazinon | 333415 | Motsuga, stone moroko | 2.3 | 3.86 | Kanazawa, J. 1978 |
| Diazinon | 333415 | Motsuga, stone moroko | 2.3 | 3.86 | Kanazawa, J. 1978 |
| Diazinon | 333415 | Motsuga, stone moroko | 2.2 | 3.86 | Kanazawa, J. 1978 |
| Diazinon | 333415 | Motsuga, stone moroko | 2.2 | 3.86 | Kanazawa, J. 1978 |
| Diazinon | 333415 | Motsuga, stone moroko | 2.2 | 3.86 | Kanazawa, J. 1978 |
| Diazinon | 333415 | Motsuga, stone moroko | 2.2 | 3.86 | Kanazawa, J. 1978 |
| Diazinon | 333415 | Motsuga, stone moroko | 2.1 | 3.86 | Kanazawa, J. 1978 |
| Diazinon | 333415 | Motsuga, stone moroko | 2.0 | 3.86 | Kanazawa, J. 1978 |
| Diazinon | 333415 | Motsuga, stone moroko | 2.0 | 3.86 | Kanazawa, J. 1978 |
| Diazinon | 333415 | Guppy | 2.2 | 3.86 | Tsuda et al. 1997 |
| Diazinon | 333415 | Guppy | 2.1 | 3.86 | Tsuda et al. 1997 |
| Diazinon | 333415 | Guppy | 2.0 | 3.86 | Tsuda et al. 1997 |
| Diazinon | 333415 | Guppy | 1.9 | 3.86 | Tsuda et al. 1997 |
| Diazinon | 333415 | Medaka, high-eyes | 2.0 | 3.86 | Tsuda et al. 1997 |
| Diazinon | 333415 | Guppy | 1.9 | 3.86 | Tsuda et al. 1997 |
| Diazinon | 333415 | Medaka, high-eyes | 1.9 | 3.86 | Tsuda et al. 1997 |
| Diazinon | 333415 | Guppy | 1.8 | 3.86 | Tsuda et al. 1997 |
| Diazinon | 333415 | Goldfish | 1.7 | 3.86 | Tsuda et al. 1997 |
| Diazinon | 333415 | Medaka, high-eyes | 1.8 | 3.86 | Tsuda et al. 1997 |
| Diazinon | 333415 | Goldfish | 1.6 | 3.86 | Tsuda et al. 1997 |
| Diazinon | 333415 | Guppy | 1.7 | 3.86 | Tsuda et al. 1997 |
| Diazinon | 333415 | Medaka, high-eyes | 1.8 | 3.86 | Tsuda et al. 1997 |
| Diazinon | 333415 | Goldfish | 1.6 | 3.86 | Tsuda et al. 1997 |
| Diazinon | 333415 | Medaka, high-eyes | 1.7 | 3.86 | Tsuda et al. 1997 |
| Diazinon | 333415 | Guppy | 1.6 | 3.86 | Tsuda et al. 1997 |
| Diazinon | 333415 | Medaka, high-eyes | 1.7 | 3.86 | Tsuda et al. 1997 |
| Diazinon | 333415 | Guppy | 1.6 | 3.86 | Tsuda et al. 1997 |
| Diazinon | 333415 | Medaka, high-eyes | 1.6 | 3.86 | Tsuda et al. 1997 |
| Diazinon | 333415 | Guppy | 1.5 | 3.86 | Tsuda et al. 1997 |
| Diazinon | 333415 | Medaka, high-eyes | 1.6 | 3.86 | Tsuda et al. 1997 |
| Diazinon | 333415 | Goldfish | 1.4 | 3.86 | Tsuda et al. 1997 |
| Diazinon | 333415 | Medaka, high-eyes | 1.6 | 3.86 | Tsuda et al. 1997 |
| Diazinon | 333415 | Goldfish | 1.4 | 3.86 | Tsuda et al. 1997 |
| Diazinon | 333415 | Medaka, high-eyes | 1.5 | 3.86 | Tsuda et al. 1997 |
| Diazinon | 333415 | White cloud mountain minnow | 1.6 | 3.86 | Tsuda et al. 1997 |
| Diazinon | 333415 | Medaka, high-eyes | 1.4 | 3.86 | Tsuda et al. 1997 |
| Diazinon | 333415 | White cloud mountain minnow | 1.3 | 3.86 | Tsuda et al. 1997 |
| Diazinon | 333415 | White cloud mountain minnow | 1.2 | 3.86 | Tsuda et al. 1997 |
| Diazinon | 333415 | Biwi lake gudgeon, goby or willow shiner | 2.4 | 3.86 | Tsuda et al. 1997 |
| Diazinon | 333415 | Biwi lake gudgeon, goby or willow shiner | 2.4 | 3.86 | Tsuda et al. 1997 |
| Diazinon | 333415 | Biwi lake gudgeon, goby or willow shiner | 2.3 | 3.86 | Tsuda et al. 1997 |
| Diazinon | 333415 | Medaka, high-eyes | 1.4 | 3.86 | Tsuda et al. 1995 |
| Chlorpyriphos | 2921882 | Sheepshead minnow | 3.3 | 4.66 | Cripe et al. 1986 |
| Chlorpyriphos | 2921882 | Sheepshead minnow | 3.2 | 4.66 | Cripe et al. 1986 |
| Chlorpyriphos | 2921882 | Sheepshead minnow | 3.0 | 4.66 | Cripe et al. 1986 |
| Chlorpyriphos | 2921882 | Sheepshead minnow | 3.0 | 4.66 | Cripe et al. 1986 |
| Chlorpyriphos | 2921882 | Sheepshead minnow | 3.0 | 4.66 | Cripe et al. 1986 |
| Chlorpyriphos | 2921882 | Sheepshead minnow | 3.0 | 4.66 | Cripe et al. 1986 |
| Chlorpyriphos | 2921882 | Sheepshead minnow | 2.9 | 4.66 | Cripe et al. 1986 |
| Chlorpyriphos | 2921882 | Sheepshead minnow | 2.9 | 4.66 | Cripe et al. 1986 |
| Chlorpyriphos | 2921882 | Sheepshead minnow | 2.8 | 4.66 | Cripe et al. 1986 |
| Chlorpyriphos | 2921882 | Sheepshead minnow | 2.8 | 4.66 | Cripe et al. 1986 |
| Chlorpyriphos | 2921882 | Sheepshead minnow | 2.8 | 4.66 | Cripe et al. 1986 |
| Chlorpyriphos | 2921882 | Sheepshead minnow | 2.8 | 4.66 | Cripe et al. 1986 |
| Chlorpyriphos | 2921882 | Sheepshead minnow | 2.8 | 4.66 | Cripe et al. 1986 |
| Chlorpyriphos | 2921882 | Sheepshead minnow | 2.6 | 4.66 | Cripe et al. 1986 |
| Chlorpyriphos | 2921882 | Sheepshead minnow | 2.6 | 4.66 | Cripe et al. 1986 |
| Chlorpyriphos | 2921882 | Sheepshead minnow | 2.6 | 4.66 | Cripe et al. 1986 |
| Chlorpyriphos | 2921882 | Sheepshead minnow | 2.5 | 4.66 | Cripe et al. 1986 |
| Chlorpyriphos | 2921882 | Sheepshead minnow | 2.4 | 4.66 | Cripe et al. 1986 |
| Chlorpyriphos | 2921882 | Sheepshead minnow | 2.4 | 4.66 | Cripe et al. 1986 |
| Chlorpyriphos | 2921882 | Sheepshead minnow | 2.4 | 4.66 | Cripe et al. 1986 |
| Chlorpyriphos | 2921882 | Sheepshead minnow | 2.3 | 4.66 | Cripe et al. 1986 |
| Chlorpyriphos | 2921882 | Sheepshead minnow | 2.2 | 4.66 | Cripe et al. 1986 |
| Chlorpyriphos | 2921882 | Sheepshead minnow | 2.1 | 4.66 | Cripe et al. 1986 |
| Chlorpyriphos | 2921882 | Sheepshead minnow | 2.1 | 4.66 | Cripe et al. 1986 |
| Chlorpyriphos | 2921882 | Sheepshead minnow | 1.8 | 4.66 | Cripe et al. 1986 |
| Chlorpyriphos | 2921882 | Sheepshead minnow | 1.8 | 4.66 | Cripe et al. 1986 |
| Chlorpyriphos | 2921882 | Sheepshead minnow | 1.6 | 4.66 | Cripe et al. 1986 |
| Chlorpyriphos | 2921882 | Inland silverside | 2.6 | 4.66 | Goodman, 1985a |
| Chlorpyriphos | 2921882 | Inland silverside | 2.3 | 4.66 | Goodman, 1985a |
| Chlorpyriphos | 2921882 | Inland silverside | 2.1 | 4.66 | Goodman, 1985a |
| Chlorpyriphos | 2921882 | Inland silverside | 2.0 | 4.66 | Goodman et al. 1985b |
| Chlorpyriphos | 2921882 | California grunion | 3.0 | 4.66 | Goodman et al. 1985b |
| Chlorpyriphos | 2921882 | California grunion | 2.8 | 4.66 | Goodman et al. 1985b |
| Chlorpyriphos | 2921882 | California grunion | 2.8 | 4.66 | Goodman et al. 1985b |
| Chlorpyriphos | 2921882 | California grunion | 2.7 | 4.66 | Goodman et al. 1985b |
| Chlorpyriphos | 2921882 | California grunion | 1.8 | 4.66 | Goodman et al. 1985b |
| Chlorpyriphos | 2921882 | California grunion | 1.8 | 4.66 | Goodman, 1985a |
| Chlorpyriphos | 2921882 | Fathead minnow | 3.2 | 4.66 | Jarvinen et al. 1983 |
| Chlorpyriphos | 2921882 | Common carp | 3.3 | 4.66 | NITE 2005 |
| Chlorpyriphos | 2921882 | Common carp | 2.4 | 4.66 | NITE 2005 |
| Chlorpyriphos | 2921882 | Guppy | 3.4 | 4.66 | Tsuda et al. 1997 |
| Chlorpyriphos | 2921882 | Guppy | 3.4 | 4.66 | Tsuda et al. 1997 |
| Chlorpyriphos | 2921882 | Guppy | 3.2 | 4.66 | Tsuda et al. 1997 |
| Chlorpyriphos | 2921882 | Guppy | 3.2 | 4.66 | Tsuda et al. 1997 |
| Chlorpyriphos | 2921882 | Medaka, high-eyes | 3.2 | 4.66 | Tsuda et al. 1997 |
| Chlorpyriphos | 2921882 | Medaka, high-eyes | 3.2 | 4.66 | Tsuda et al. 1997 |
| Chlorpyriphos | 2921882 | Goldfish | 2.9 | 4.66 | Tsuda et al. 1997 |
| Chlorpyriphos | 2921882 | Goldfish | 2.9 | 4.66 | Tsuda et al. 1997 |
| Chlorpyriphos | 2921882 | Goldfish | 2.8 | 4.66 | Tsuda et al. 1997 |
| Chlorpyriphos | 2921882 | Common carp | 2.7 | 4.66 | Tsuda et al. 1992 |
| Chlorpyriphos | 2921882 | Common carp | 2.7 | 4.66 | Tsuda et al. 1992 |
| Chlorpyriphos | 2921882 | Common carp | 2.6 | 4.66 | Tsuda et al. 1992 |
| Chlorpyriphos | 2921882 | Common carp | 2.5 | 4.66 | Tsuda et al. 1992 |
| Chlorpyriphos | 2921882 | Common carp | 2.5 | 4.66 | Tsuda et al. 1992 |
| Chlorpyriphos | 2921882 | Rainbow trout | 3.5 |  | Adolfsson-Erici et al. 2012 |
| ^14^C-Pyrene | 129000 | Guppy | 3.0 | 4.93 | Carlson et al. 1979 |
| ^14^C-Pyrene | 129000 | Guppy | 3.4 | 4.93 | Carlson et al. 1979 |
| ^14^C-Pyrene | 129000 | Guppy | 3.4 | 4.93 | Carlson et al. 1979 |
| ^14^C-Pyrene | 129000 | Guppy | 3.1 | 4.93 | Carlson et al. 1979 |
| ^14^C-Pyrene | 129000 | Guppy | 3.1 | 4.93 | Carlson et al. 1979 |
| ^14^C-Pyrene | 129000 | Guppy | 3.1 | 4.93 | Carlson et al. 1979 |
| ^14^C-Pyrene | 129000 | Sheepshead minnow | 2.2 | 4.93 | Jonsson et al. 2004 |
| ^14^C-Pyrene | 129000 | Sheepshead minnow | 2.0 | 4.93 | Jonsson et al. 2004 |
| ^14^C-Pyrene | 129000 | Sheepshead minnow | 1.7 | 4.93 | Jonsson et al. 2004 |
| ^14^C-Pyrene | 129000 | Sheepshead minnow | 1.7 | 4.93 | Jonsson et al. 2004 |
| ^14^C-Simazine | 122349 | Common carp | 1.1 | 2.4 | CERI 1992 |
| ^14^C-Simazine | 122349 | Common carp | 0.4 | 2.4 | CERI 1992 |
| ^14^C-Simazine | 122349 | Willow shiner | 1.4 | 2.4 | Tsuda et al. 1992 |
| ^14^C-Simazine | 122349 | Willow shiner | 1.3 | 2.4 | Tsuda et al. 1992 |
| ^14^C-Simazine | 122349 | Willow shiner | 1.3 | 2.4 | Tsuda et al. 1992 |
| ^14^C-Simazine | 122349 | Willow shiner | 1.1 | 2.4 | Tsuda et al. 1992 |
| ^14^C-Simazine | 122349 | Willow shiner | 1.1 | 2.4 | Tsuda et al. 1992 |
| ^14^C-LHC | N/A | Rainbow trout | 1.9 | 3.4 | Schlechtriem et al. 2016 |
| Methoxychlor | 72435 | Rainbow trout | 3.2 |  | Bischof 2018 |

**S2.2 References for studies reported in Table S2.1 and S2.2**

Adolfsson‐Erici M, Åkerman G, McLachlan MS (2012) Measuring bioconcentration factors

in fish using exposure to multiple chemicals and internal benchmarking to correct for growth

dilution. Environ Toxicol Chem 31: 1853–1860

Arnot JA, Gobas FAPC (2006) A review of bioconcentration factor (BCF) and bioaccumulation factor (BAF) assessments for organic chemicals in aquatic organisms. Environ Rev 14: 257–297

Bischof I (2018) Primary fish hepatocytes as in vitro test system to study the metabolism of xenobiotics in fish (Doctoral dissertation). Vetsuisse-Fakultät, Universität Bern

Böhm L, Düring RA, Bruckert HJ, Schlechtriem C (2017) Can solid-phase microextraction replace solvent extraction for water analysis in fish bioconcentration studies? Environ Toxicol Chem 36: 2887-2894

Carlson AR, Kosian PA (1987) Toxicity of chlorinated benzenes to fathead minnows (*Pimephales promelas*). Arch Environ Contam Toxicol 16: 129-135

Carlson RM, Oyler AR, Gerhart EH, Caple R, Welch KJ, Kopperman HL, Bodenner D, Swanson D (1979) Implications to aquatic environment of polynuclear aromatic hydrocarbons liberated from northern great plains coal. EPA-600/3-79-093: 156 p. U.S.EPA, Duluth, MN, USA

CERI (1992) Biodegradation and bioaccumulation data of existing chemicals based on the CSCL Japan, Japan Chemical Industry Ecology [online]. Toxicology and Information Center, Chemicals Evaluation and Research Institute. [cited in Arnot and Gobas 2006].

Chaisuksant Y, Yu Q, Connell DW (1997) Bioconcentration of bromo- and chlorobenzenes by fish (*Gambusia affinis*). Water Research 31: 61-68

Cripe GM, Hansen DJ, MaCauley SF, Forester J (1986) Effects of diet quantity on sheepshead minnows (*Cyprinodon variegatus*) during early life-stage exposures to chlorpyrifos. In: T.M. Poston and R. Purdy (Eds.), Aquatic Toxicology and Environmental Fate, 9th Volume, ASTM STP 921: p. 450-460. Philadelphia, PA, USA

Fox K, Zauke GP, Butte W (1994) Kinetics of bioconcentration and clearance of 28 polychlorinated biphenyl congeners in zebrafish (*Brachydanio rerio*). Ecotox Environ Safety 28: 99-109

Galassi S, Calamari D (1983) Toxicokinetics of 1,2,3 and 1,2,4 trichlorobenzenes in early life stages of *Salmo gairdneri*. Chemosphere 12:1599-1603

Goodman LR, Hansen DJ, Cripe G, Middaugh DP, Moore JC (1985) A new early life-stage toxicity test using the california grunion (*Leuresthes tenuis*) and results with chlorpyrifos. Ecotoxicol Environ Saf 10: 12-21

Goodman LR, Hansen DJ, Middaugh DP, Cripe GM, Moore JC (1985) Method for early life-stage toxicity tests using three atherinid fishes and results with chlorpyrifos. In: R.D. Cardwell, R. Purdy and R.C. Bahner (Eds.), Aquatic Toxicology and Hazard Assessment, 7th Symposium, ASTM STP 854. p. 145-154. Philadelphia, PA, USA

Jarvinen AW, Nordling BR, Henry ME (1983) Chronic toxicity of dursban (Chlorpyrifos) to the fathead minnow (*Pimephales promelas*) and the resultant acetylcholinesterase inhibition. Ecotoxicol Environ Saf 7: 423-434

Jimenez BD, Cirmo CP, McCarthy JF (1987) Effects of feeding and temperature on uptake, elimination and metabolism of benzo(a)pyrene in the bluegill sunfish (*Lepomis macrochirus*). Aquat Toxicol 10: 41-57

Jonsson G, Bechmann RK, Bamber SD, Baussant T (2004) Bioconcentration, biotransformation, and elimination of polycyclic aromatic hydrocarbons in sheepshead minnows (*Cyprinodon variegatus*) exposed to contaminated seawater. Environ Toxicol Chem 23: 1538-1548

Kanazawa J (1978) Bioconcentration ratio of diazinon by freshwater fish and snail. Bull Environ Contam Toxicol 20: 613-617

Kosian P, Lemke A, Studders K, Veith G (1981) The precision of the ASTM bioconcentration test. EPA 600/3-81-022, 20 p. US EPA, Duluth, MN, USA

National Institute of Technology and Evaluation (2005) Biodegradation and bioconcentration of existing chemical substances under the chemical substances control Law, , <http://www.nite.go.jp/index-e.html> (cited in Arnot and Gobas 2006).

Opperhuizen A, Schrap SM (1987) Relationships between aqueous oxygen concentration and uptake and elimination rates. Environ Toxicol Chem 6: 335–342

Opperhuizen A, Voors PI (1987) Bioconcentration kinetics of 2,4,5- tri- and 3,3',4,4'-tetrachlorobiphenyl and 2,4,5- tri- and 3,3',4,4'-tetrachlorodiphenylether in fish. Chemosphere 16: 2379-2388

Schlechtriem C, Bischof I, Atorf C, Bergendahl E, Seymour P, Whalley P (2016) Development of a regulatory testing procedure to study the metabolism of pesticides in farmed fish. Pest Manag Sci, 72: 362-370

Tsuda T, Aoki S, Inoue T, Kojima M (1995) Accumulation and excretion of diazinon, fenthion and fenitrothion by killifish: Comparison of individual and mixed pesticides. Water Res. 29: 455-458

Tsuda T, Aoki S, Kojima M, Fujita T (1992) Accumulation and excretion of pesticides used in golf courses by carp (*Cyprinus carpio*) and willow shiner (*Gnathopogon caerulescens*). Comp Biochem Physiol C 101: 63-66

Tsuda T, Kojima M, Harada H, Nakajima A, Aoki S (1997) Relationships of bioconcentration factors of organophosphate pesticides among species of fish. Comp Biochem Physiol C, 116: 213-218

**Supplementary material (Part 3)**

**3.1 Results of linear regression**

**Figure 5; all fish data**

Fish = 0.251 + (0.792 * Hyalella)

N = 148

R = 0.829 **Rsqr = 0.687** Adj Rsqr = 0.685

**Standard Error of Estimate = 0.540**

**Coefficient Std. Error t P**

Constant 0.251 0.136 1.845 0.067

Hyalella 0.792 0.0442 17.916 <0.001

Analysis of Variance:

**DF SS MS F P**

Regression 1 93.662 93.662 320.985 <0.001

Residual 146 42.602 0.292

Total 147 136.264 0.927

Normality Test (Shapiro-Wilk) Passed (P = 0.131)

Power of performed test with alpha = 0.050: 1.000

**Figure 6a; rainbow trout**

Trout = 1.220 + (0.638 * Hyalella)

N = 9

R = 0.878 **Rsqr = 0.771** Adj Rsqr = 0.738

**Standard Error of Estimate = 0.529**

**Coefficient Std. Error t P**

Constant 1.220 0.481 2.537 0.039

Hyalella 0.638 0.131 4.854 0.002

Analysis of Variance:

**DF SS MS F P**

Regression 1 6.596 6.596 23.557 0.002

Residual 7 1.960 0.280

Total 8 8.556 1.069

Normality Test (Shapiro-Wilk) Passed (P = 0.245)

Power of performed test with alpha = 0.050: 0.918

**Figure 6b; common carp**

Carp = 0.428 + (0.749 * Hyalella)

N = 19

R = 0.801 **Rsqr = 0.641** Adj Rsqr = 0.620

**Standard Error of Estimate = 0.596**

**Coefficient Std. Error t P**

Constant 0.428 0.411 1.041 0.312

Hyalella 0.749 0.136 5.511 <0.001

Analysis of Variance:

**DF SS MS F P**

Regression 1 10.784 10.784 30.371 <0.001

Residual 17 6.037 0.355

Total 18 16.821 0.935

Normality Test (Shapiro-Wilk) Passed (P = 0.182)

Power of performed test with alpha = 0.050: 0.993

**Figure 6c; guppy**

Guppy = 0.0593 + (0.912 * Hyalella)

N = 22

R = 0.959 **Rsqr = 0.920** Adj Rsqr = 0.916

**Standard Error of Estimate = 0.307**

**Coefficient Std. Error t P**

Constant 0.0593 0.190 0.311 0.759

Hyalella 0.912 0.0602 15.131 <0.001

Analysis of Variance:

**DF SS MS F P**

Regression 1 21.551 21.551 228.961 <0.001

Residual 20 1.883 0.0941

Total 21 23.434 1.116

Normality Test (Shapiro-Wilk) Passed (P = 0.886)

Power of performed test with alpha = 0.050: 1.000
